# Self-Immolative Polymers: An Emerging Class of Degradable Materials with Distinct Disassembly Profiles

**DOI:** 10.1021/jacs.1c11410

**Published:** 2021-12-13

**Authors:** Omri Shelef, Samer Gnaim, Doron Shabat

**Affiliations:** School of Chemistry, Raymond and Beverly Sackler Faculty of Exact Sciences, Tel-Aviv University, Tel Aviv 69978, Israel

## Abstract

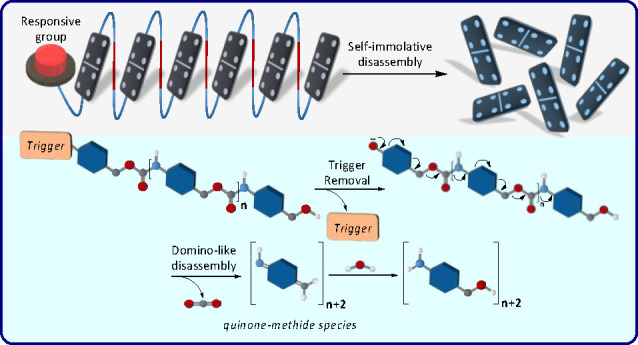

Self-immolative polymers are an emerging class of macromolecules with distinct disassembly profiles that set them apart from other general degradable materials. These polymers are programmed to disassemble spontaneously from head to tail, through a domino-like fragmentation, upon response to extremal stimuli. In the time since we first reported this unique type of molecule, several groups around the world have developed new, creative molecular structures that perform analogously to our pioneering polymers. Self-immolative polymers are now widely recognized as an important class of stimuli-responsive materials for a wide range of applications such as signal amplification, biosensing, drug delivery, and materials science. The quinone-methide elimination was shown to be an effective tool to achieve rapid domino-like fragmentation of polymeric molecules. Thus, numerous applications of self-immolative polymers are based on this disassembly chemistry. Although several other fragmentation reactions achieved the function requested for sequential disassembly, we predominantly focused in this Perspective on examples of self-immolative polymers that disassemble through the quinone-methide elimination. Selected examples of self-immolative polymers that disassembled through other chemistries are briefly described. The growing demand for stimuli-responsive degradable materials with novel molecular backbones and enhanced properties guarantees the future interest of the scientific community in this unique class of polymers.

## Introduction and Historical Overview

Stimuli-responsive materials are a promising type of smart molecular compounds, with properties that can be significantly changed in a controlled fashion upon response to applied stimuli. About 14 years ago our group developed a distinct class of such molecular compounds that we termed as self-immolative polymers.^1^ These polymers are programmed to disassemble spontaneously from head to tail, through a domino-like fragmentation, upon response to external stimuli. Since we developed this type of macromolecules, the field of self-immolative polymers has grown beyond our expectations. Numerous groups from around the world have reported new, creative molecular structures, which perform in manners analogous to our original concept of self-immolative polymers. Currently, self-immolative polymers are widely recognized as an important emerging class of stimuli-responsive materials for a wide range of applications.^2,3^ In this Perspective, we first give a historical overview of the field and then focus on selected examples that, in our view, reflect the unique significance of self-immolative polymers. Finally, we describe the recent developments of new molecular structures that cleverly achieve the distinctive functions demanded of self-immolative polymers.

Depolymerization reactions of polymers with low ceiling temperatures, such as poly(olefin sulfone) and poly(phthalaldehyde), were described by Ito and others, long before we reported the concept of self-immolative polymers.^4−6^ However, there is a fundamental difference between such depolymerization processes and the disassembly pathway of self-immolative polymers. The disassembly of a self-immolative polymer is initiated upon removal of a specific masking group at the polymer terminus and occurs from head to tail through a domino-like manner, while depolymerization of polymers with low ceiling temperatures is initiated at various locations along the polymer backbone by radiation or photoacids. Thus, the level of control on the disassembly of self-immolative polymers is more modular and versatile.

**Figure 1:**
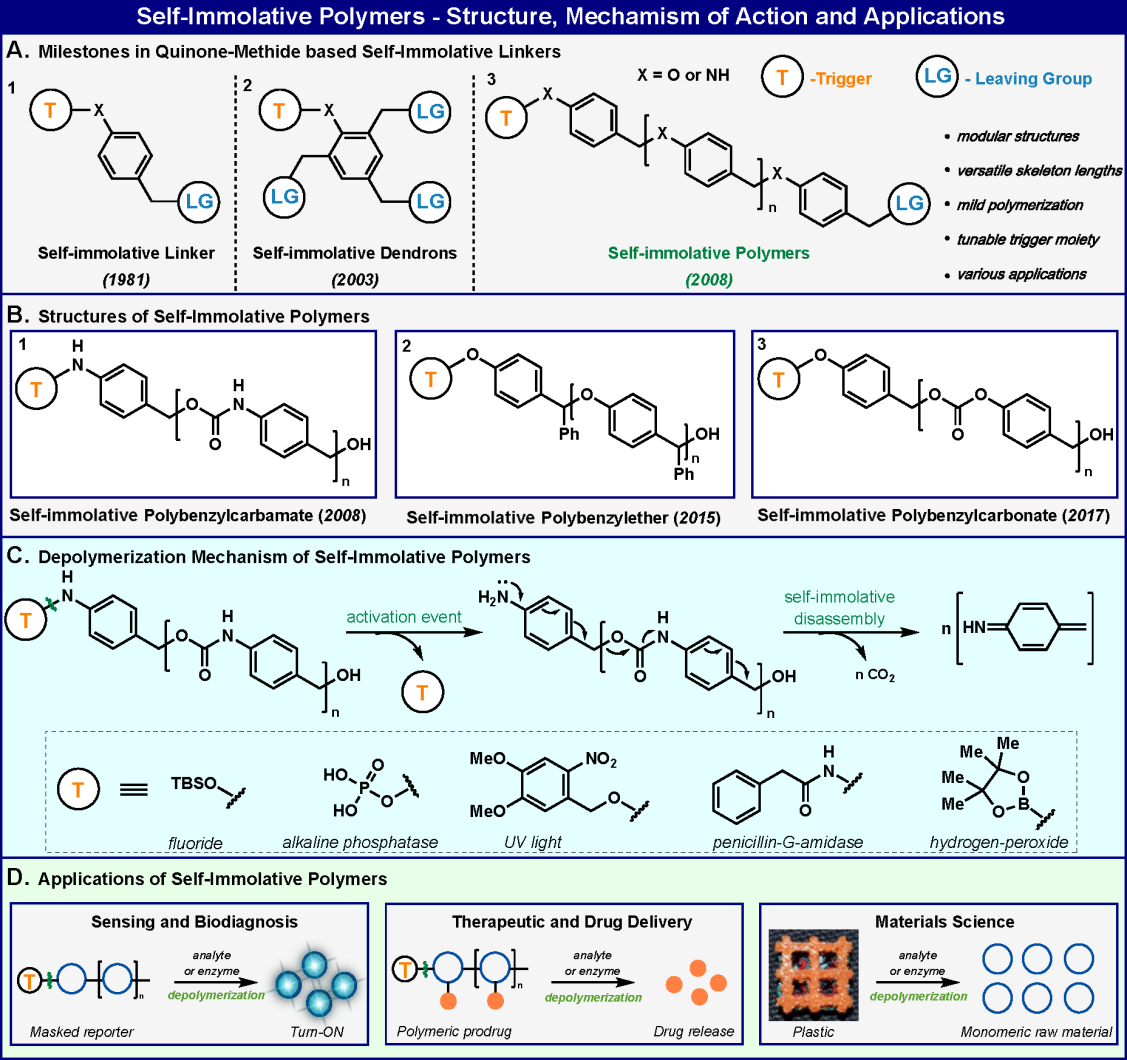
Self-immolative polymers: historical viewpoint, molecular structures, disassembly mechanism, and applications. Image in (D) reproduced with permission from ref (43). Copyright 2015 John Wiley and Sons.

Intriguingly, the terminology “self-immolative”, in regard to chemistry, was used for the first time by Mislow in 1965, to describe the chirality loss of a reagent in asymmetric synthesis.^7^ In 1981, Katzenellenbogen reported the use of a novel connector linkage, based on 4-aminobenzyl alcohol, as a self-immolative linker (Figure 1A.1).^8^ This linker molecule is used to connect a specific substrate to a target molecule via stable chemical bonds. Removal of the substrate, by an external stimulus, resulted in the formation of an aniline derivative, which rapidly underwent 1,6-elimination to form aza-quinone-methide and to release the target molecule. Such linkers structurally “sacrifice” themselves in order to implement their designated function. Years later, these earlier discoveries inspired us^9^ and others^10,11^ to explore related branched molecular linkers (Figure 1A.2) and led to the development of novel macromolecules, known today as self-immolative dendrimers. Such dendrimers can undergo sequential disassembly from their focal terminus to the periphery upon removal of a responsive group by an external stimulus. As a result, these treelike molecules can act as efficient molecular amplifiers through release of multiple peripheral end groups upon a single cleavage event, which takes place at the focal point.^12,13^

The disassembly mechanism of most self-immolative linkers and dendrimers described to date is predominantly based on the quinone-methide elimination. This elimination is a valuable tool for achieving various important molecular functions.^14^ In 2008, we took advantage of the quinone-methide elimination reaction to design a macromolecular scaffold for the first example of the synthesis of self-immolative polymers (Figure 1A.3).^1^ The polymer was prepared by polymerization of an appropriate monomer to form a polybenzylcarbamate backbone, and the head terminus was then capped with a specific responsive group that acts as a trigger (Figure 1B.1). Two additional examples of self-immolative polymers that employ the quinone-methide elimination in their disassembly pathway were subsequently reported. The Phillips group reported the design and synthesis of a self-immolative polymer with a poly(benzyl ether) backbone^15^ (Figure 1B.2), and our group prepared a self-immolative polymer with a poly(benzylcarbonate) backbone^16^ (Figure 1B.3). The disassembly of the self-immolative polymer with a poly(benzylcarbamate) backbone occurs upon removal of the head trigger by an external stimulus, which releases an aniline functional group that then undergoes sequential fragmentation through aza-quinone-methide elimination and decarboxylation reactions (Figure 1C). Analogous disassembly mechanisms can be sketched for self-immolative polymers with poly(benzyl ether) and poly(benzylcarbonate) backbones. Various responsive groups have been used to trigger degradation by enzymes, chemical analytes, or UV light. Self-immolative polymers have been demonstrated to be useful for a vast range of applications, including molecular amplification and biosensing,^17^ drug delivery,^18,19^ and materials science^20^ (Figure 1D).

The quinone-methide elimination is an effective tool to achieve rapid domino-like fragmentation of polymeric molecules.^14^ Thus, numerous examples of self-immolative polymers are based on this disassembly chemistry. Although several other rapid fragmentation reactions have been shown to induce sequential disassembly, we predominantly focus here on examples of self-immolative polymers that spontaneously disassemble through the quinone-methide elimination. Selected examples of self-immolative polymers that disassembled through chemistries other than quinone-methide elimination are described in the last section of this Perspective.

## Signal Amplification and Diagnostic Applications

**Figure 2:**
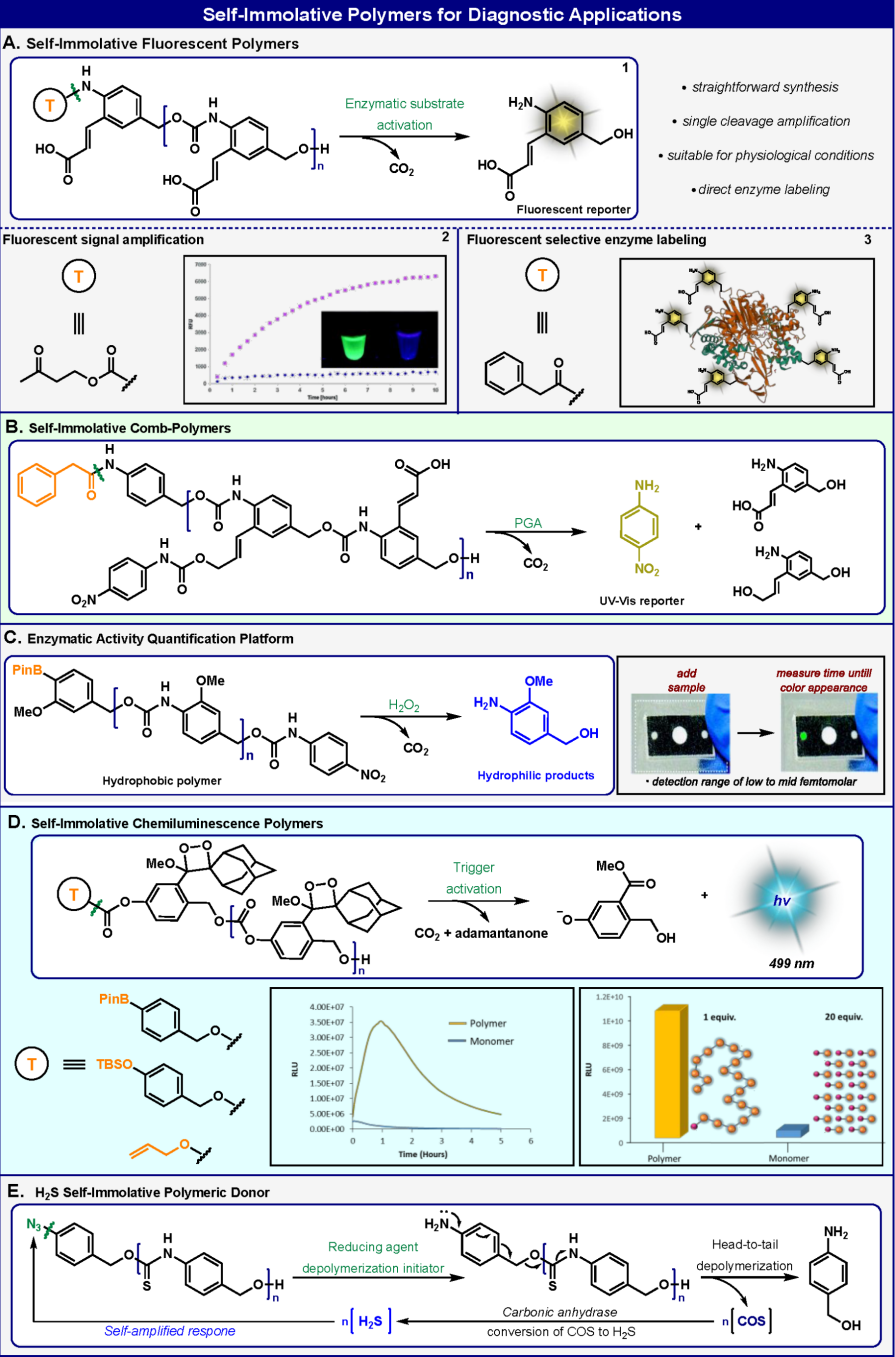
Applications of self-immolative polymers as optical probes for sensing and labeling.

The first example of self-immolative polymers was demonstrated for an application that employs a signal amplification technique (Figure 2A.1). To achieve this function, we designed a monomeric building block, composed of an aniline molecule with an *o*-acrylate substituent.^1^ This aniline derivative produces green fluorescent emission, when its amino functional group is free. However, once this monomeric building block is polymerized to generate a poly(benzylcarbamate) backbone, the fluorescence of the aniline is almost completely quenched. The polymer was capped with 4-hydroxy-2-butanone as a head trigger, which is designed for removal through a β-elimination reaction catalyzed by the protein bovine serum albumin (BSA). Incubation of this self-immolative polymer with BSA initiates the domino-like disassembly of the polymer molecule to its aniline monomeric units. As a result, a green fluorescence signal gradually increases in intensity correlated to the number of monomers released (Figure 2A.2).

In a subsequent report, our group showed that this type of self-immolative polymer can be applied as an activity-based probe for labeling of proteins with catalytic activity.^21^ Cleavage of the head trigger by the protein of interest leads to selective labeling of the protein by the reaction of nucleophilic residues on the protein with the quinone-methide units released by the self-immolative polymer disassembly process. This reaction converts the released quinone-methide species to a fluorescent aniline label. We demonstrated that a self-immolative polymer equipped with a phenyl-acetamide head trigger effectively labeled the enzyme penicillin-G-amidase (Figure 2A.3).

Replacing the *o*-acrylate substituent in the aniline monomer with a vinylogous *o*-hydroxybenzyl substituent produces a molecular unit that can undergo 1,6-elimination to release a reporter group. Such an aniline monomer was used as a unique building block to prepare self-immolative comb polymers.^22^ Removal of the head trigger initiates the polymer disassembly into its building blocks, followed by spontaneous release of the reporters from each monomer. A water-soluble version of a self-immolative comb polymer was shown to undergo an efficient domino-like disassembly and release of 4-nitroaniline side groups in response to the protease penicillin-G-amidase under physiological conditions (Figure 2B). The released 4-nitroaniline molecule produces a measurable optical signal that can be used for diagnostic purposes. The use of drug molecules instead of the optically active reporter units yields a self-immolative comb-polymeric drug delivery system that can selectively release a high payload of drug upon a cleavage event by a specific enzyme.

The Phillips group used a self-immolative poly(benzylcarbamate) oligomer to develop a strategy for quantifying active enzyme analytes in a paper-based device^23^ (Figure 2C). A self-immolative hydrophobic oligomer, equipped with a pinacol-boronate head trigger, was used to amplify a signal for the detection event. The amplification occurred upon head to tail depolymerization in response to the hydrogen peroxide that is generated during the detection event. The principle of the assay is based on the rapid conversion of a large hydrophobic oligomer into small-molecule hydrophilic products, a task achieved by the disassembly properties of the self-immolative polybenzylcarbamate oligomer. Reagents for the detection of specific substrates were incorporated into the device to provide selective detection of the target enzyme analytes.

Chemiluminescence is a powerful tool for the development of sensitive diagnostic assays.^24,25^ Our group reported the development of self-immolative poly(benzylcarbonates) that generate a distinct chemiluminescence diagnostic signal^16^ (Figure 2D). This polymer links a chemiluminescence turn-ON property with a self-immolative disassembly mechanism. Chemiluminescence occurs upon head-to-tail disassembly after a triggering event caused by an external stimulus, generated through various responsive groups. The polymer building block unit combines the chemiexcitation mechanism of Schaap’s adamantylidene-dioxetane^26^ and the quinone-methide elimination, which is used for the self-immolative disassembly of the polymer. The chemiexcitation of each phenoxy-dioxetane unit occurred as a result of a phenolate formation during the polymer disassembly. Electron transfer from the phenolate anion to the σ* orbital of the dioxetane–peroxide bond leads to the release of excited benzoate species that decays to its ground state through release of a photon. The amplification of intensity and the duration of the light emission by these poly(benzylcarbonate) self-immolative polymers correlated with their lengths.

Signal amplification molecular systems are of obvious importance for various diagnostic assays.^27−32^ Matson and co-workers reported the synthesis of a self-immolative polymer with a poly(benzylthiourethane) backbone suitable for diagnostic use^33^ (Figure 2E). The polymer head was capped with an aryl-azide responsive group as a trigger that could be activated by hydrogen sulfide. The polymer backbone was disassembled through consecutive quinone-methide eliminations to release carbonyl-sulfide. The released carbonyl-sulfide was converted into hydrogen sulfide by the enzyme carbonic anhydrase. As a result, the polymer degradation was enhanced through the generated amplified response.

## Materials Science Applications

**Figure 3:**
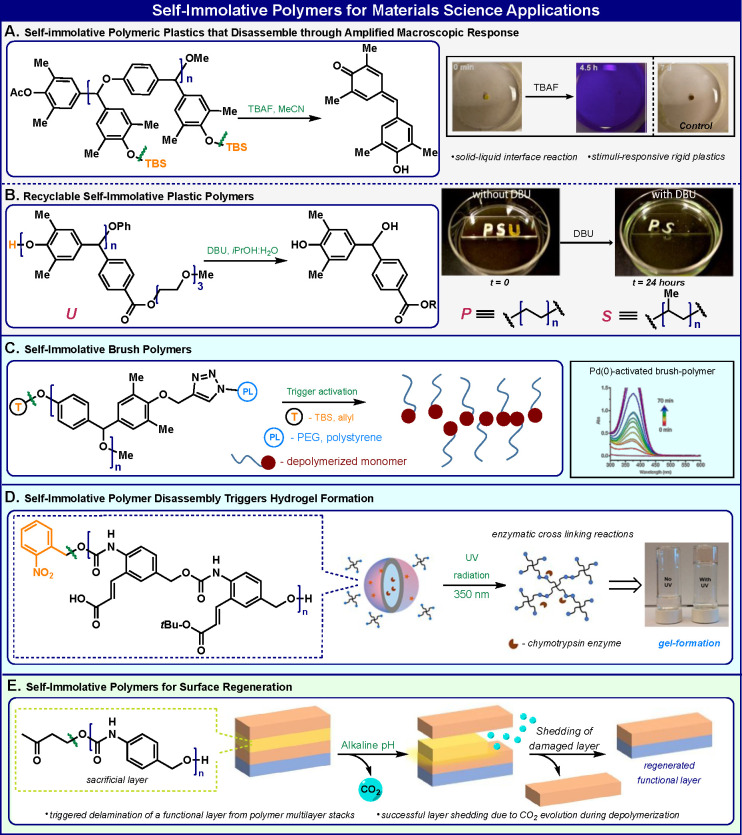
Selected examples of self-immolative polymers applications in materials science. Image (B) reproduced with permission from ref (34). Copyright 2015 The Royal Society of Chemistry.

The unique disassembly properties of self-immolative polymers make them ideal macromolecules for materials science applications that require degradable compounds. The Phillips group has significantly contributed to this field with milestone demonstrations of such materials. In a seminal example, they synthesized a new type of poly(benzyl ether) self-immolative polymer with multiple responsive groups incorporated at the benzylic positions of each building block^15^ (Figure 3A). The polymers were used to prepare stimuli-responsive, rigid plastics that amplify responses to specific signals at the solid–liquid interface. Solid-state plastic disks, prepared from these polymers, selectively depolymerize upon exposure to a specific stimulus. The disassembly of the polymer releases purple-blue quinone-methide monomeric units. In subsequent work, Phillips and co-workers demonstrated synthetic strategies for modifying the poly(benzyl ether) self-immolative polymers in order to obtain the desired properties in plastics^34^ (Figure 3B). Plastics were designed to allow selective, room temperature, and self-immolative depolymerization into their recyclable monomers when the plastic is no longer needed.

Zhang and co-workers have also used the poly(benzyl ether) backbone to prepare self-immolative brush polymers by grafting azide-terminated side chains onto an alkyne-bearing scaffold^35^ (Figure 3C). In order to graft a brushlike structure, two types of azide-terminated polymers, poly(styrene) and poly(ethylene glycol), were used as side chains on the polymer backbone. Upon exposure to a specific reagent, these brush polymers undergo self-immolative disassembly through sequential quinone-methide eliminations to yield individual side chains.

Thayumanavan’s group reported the use of a self-immolative polymer with a polybenzylcarbamate backbone bearing anionic functional groups to form vesicles upon complexation with a cationic counterpart polymer^36^ (Figure 3D). These nanoparticles were formulated in aqueous solution and were able to entrap and accommodate hydrophilic guest molecules, including enzymes. Removal of the head trigger by UV light initiates the head to tail depolymerization of the self-immolative polymer molecules and causes the vesicles to disassemble and releases their contents. The released enzyme then reacts with additional released components to cause a phase change to a self-supporting hydrogel. This example demonstrated a general approach to the use of a self-immolative disassembly of a polymeric molecule to construct a different species. In another approach to enable surface generation, Lienkamp and co-workers used a self-immolative polymer with a poly(benzylcarbamate) backbone to prepare a functional surface in a multilayer device (Figure 3E).^37^ The polymer head was capped with 4-hydroxy-2-butanone as a responsive group that can be removed under basic conditions through a β-elimination reaction. The polymer disassembly enables self-regeneration of the functional layer in the polymer multilayer.

## Therapeutic and Controlled-Release Applications

**Figure 4:**
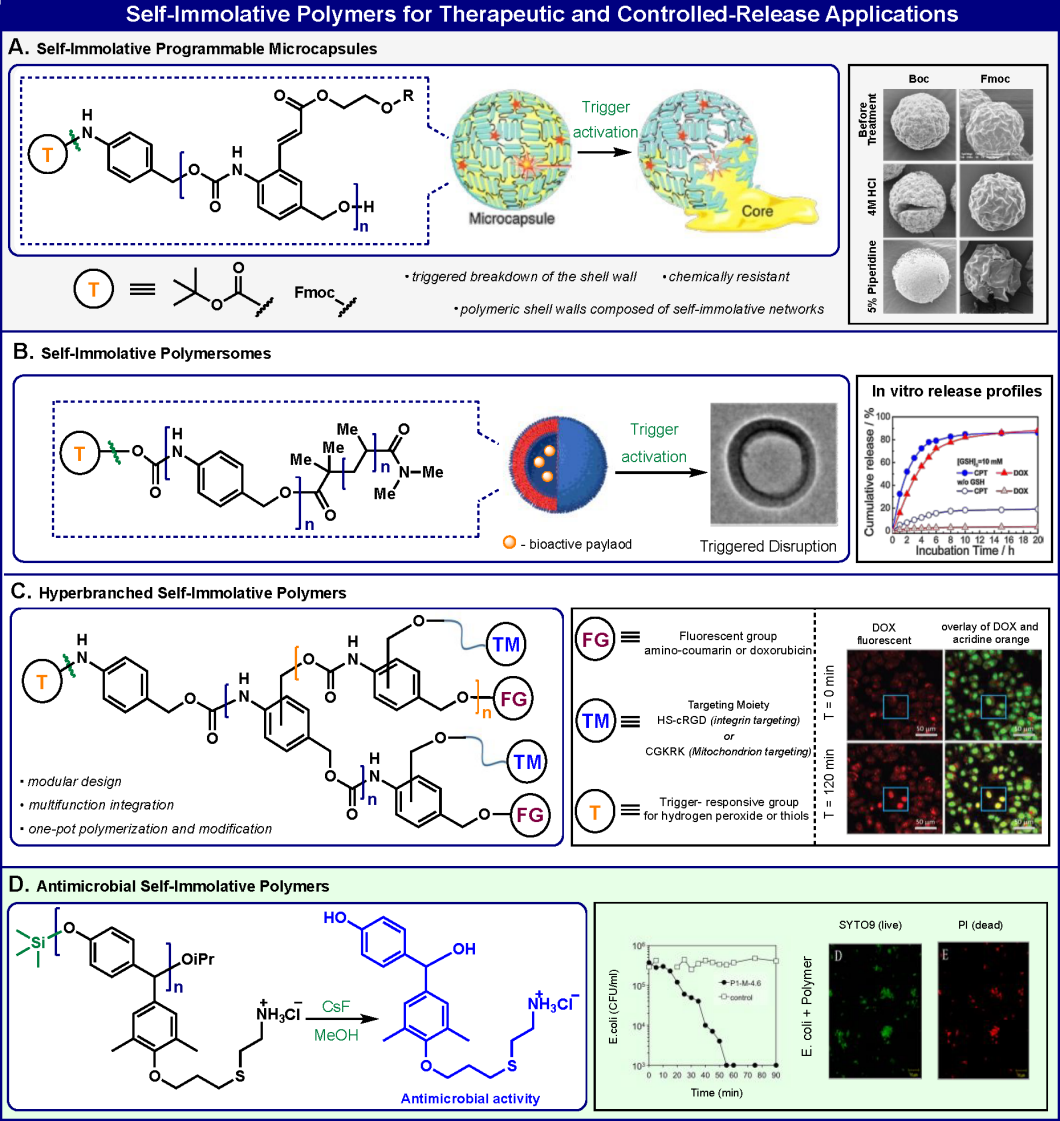
Applications of self-immolative polymers in therapeutic science and controlled release.

Moore and co-workers used our polybenzylcarbamate self-immolative polymers to prepare programmable microcapsules that can release their contents in response to an external stimulus^38^ (Figure 4A). Microcapsules were synthesized via an interfacial polymerization method between the hydroxyl side group of the self-immolative polymers to isocyanates and 1,4-butanediol. The capsules’ shell morphology was examined to confirm that rupture of the shell wall was indeed, the programmable mechanism of release. The capsules were shown to release their core contents upon exposure to a specific reagent that can remove the chemical protecting group of the polymer head. These types of “on-demand” chemical systems could be useful in diverse areas of controlled-release techniques such as drug delivery applications.

The Liu group prepared amphiphilic block copolymers by linking a poly(benzylcarbamate) self-immolative polymer with a hydrophilic polymeric block (Figure 4B). These amphiphilic block copolymers self-assemble into self-immolative polymersomes.^39^ Removal of the terminal capping groups by visible light, UV light, or reductive conditions initiates the disassembly of the polymersomes into water-soluble small molecules and hydrophilic blocks. Several controlled-release examples with various contents, such as the anticancer drugs doxorubicin and camptothecin, were demonstrated. In a subsequent work, the same group reported the preparation of a hyperbranched self-immolative polymer with a poly(benzylcarbamate) backbone^40^ (Figure 4C). The monomeric unit of this polymer is composed of a building block, which was used for the synthesis of self-immolative dendrimers. A polycondensation reaction between hydroxybenzyl substituents and isocyanates of the monomeric building block afforded the desired hyperbranched polybenzylcarbamate polymer, which was then capped with various substrates as head triggers. Fluorescent dyes or drug molecules were incorporated at the dendritic end sites. Exposure of these molecules to an analyte of interest initiates the domino-like disassembly of the hyperbranched polymer through sequential quinone-methide eliminations. As a result, multiple end groups are released upon a single cleavage event of the head trigger. Structurally, the molecular configuration of this type of polymer is based on a combination of the molecular structures of self-immolative dendrimers and self-immolative polymers.

The Palermo group reported the first example of self-immolative polymers that have antibacterial activity^41^ (Figure 4D). They synthesized a self-immolative polymer with a poly(benzyl ether) backbone and ammonium cationic side groups. The polymer head is capped with a silyl ether protecting group as a responsive substrate for a fluoride ion. The intact polycationic self-immolative polymer is highly stable in solution, but upon exposure to fluoride ions, the responsive group is removed and the polymeric molecules spontaneously disassemble into the component monomers. The self-immolative polymers bearing ammonium cationic groups have potent bactericidal activity against *Escherichia coli*. Remarkably, the antibacterial potency of the cationic monomer units, obtained through the domino-like disassembly, is largely retained, but their hemolytic toxicity is substantially reduced.

## Self-Immolative Polymers Composed of Various Polymeric Backbones

The quinone-methide disassembly chemistry of self-immolative polymers, reported by our group, inspired several other groups to develop new disassembly chemistries. In this section, we describe selected examples of self-immolative polymers, composed of various backbones, that disassemble through new disassembly mechanisms or through mechanistic pathways that involve quinone-methide elimination in combination with additional fragmentation reactions.

**Figure 5:**
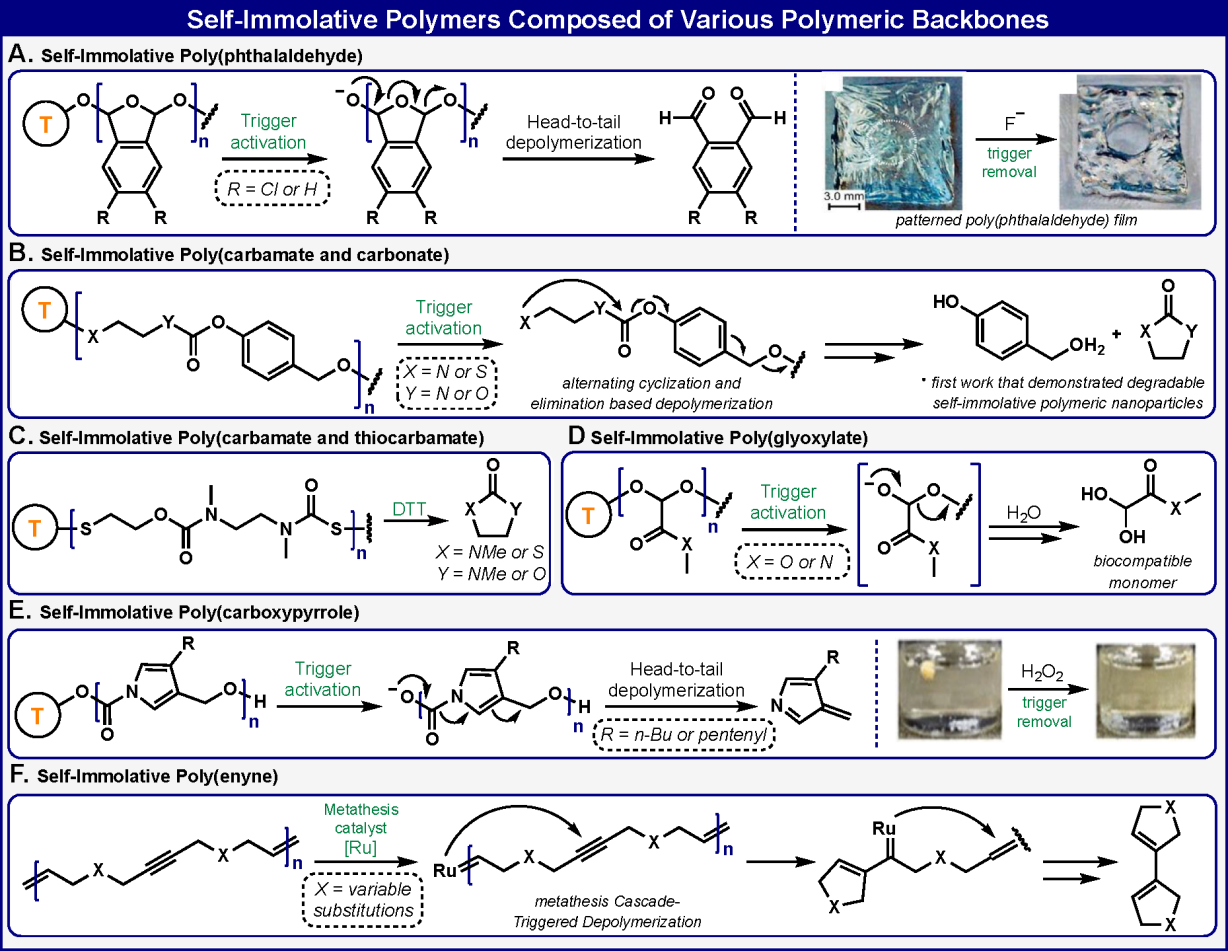
Selected examples of self-immolative polymers composed of various polymeric backbones.

In 2010, the Phillips group developed self-immolative polymers that disassemble from head to tail by a chemistry different from the quinone-methide elimination.^42^ They synthesized poly phthalaldehyde polymers, from phthalaldehyde monomeric units, using anionic polymerization conditions (Figure 5A). The obtained self-immolative poly phthalaldehyde was fabricated to form a plastic. Different polymers within the plastic were equipped with different responsive groups and, thus, were capable of responding to different signals. Removal of a specific responsive group initiated the head-to-tail disassembly of the corresponding polymer. The process of this specific depolymerization resulted in rapid alterations of the physical features of the plastic to a degree that corresponded to the length of the responsive polymer. In subsequent work, the same group synthesized end-capped poly(4,5-dichlorophthalaldehyde) self-immolative polymers.^43^ These unique polymers can autonomously amplify macroscopic changes in materials in response to a specific stimulus.

The group of Gillies prepared self-immolative polymers that can disassemble through alternating cyclization and quinone-elimination reactions^44^ (Figure 5B). These polymers are composed of *N,N′-*dimethylethylenediamine and 4-hydroxybenzyl alcohol molecules linked by carbamate linkages to form a monomeric building block. To demonstrate the degradability of these polymers under biologically relevant conditions, poly(ethylene oxide) was introduced as an end cap via an ester linkage to produce an amphiphilic block copolymer. This copolymer was assembled into nanoparticles that were capable of encapsulating and subsequently releasing a fluorescent dye in aqueous solution through a slow domino-like disassembly process.

Gillies and co-workers have also reported a new type of self-immolative polymer backbone based on *N,N′-*dimethylethylenediamine and 2-mercaptoethanol linked by carbamate and thiocarbamate bonds^45^ (Figure 5C). A disulfide end cap was incorporated such that cleavage under reducing conditions unmasked the thiol functional group of 2-mercaptoethanol. This polymer disassembles from head to tail through sequential, alternating cyclization reactions to release molecules with five-membered rings. In additional elegant work, the Gillies group demonstrated how polyglyxoylates can serve as a new and versatile class of self-immolative polymers^46^ (Figure 5D). The commercially available monomer ethyl glyoxylate was polymerized to generate a poly(ethyl glyoxylate). The polymer terminus head was sealed with 6-nitroveratryl carbonate as a responsive triggering substrate. Upon irradiation with UV light, the polymer undergoes head to tail disassembly to generate ethanol and the metabolic intermediate glyoxylic acid hydrate. These new self-immolative polymers are particularly attractive, as their component monomers can be derived not only from petroleum-based sources but also from renewable resources. In addition, such polymers disassemble to release component monomers that are nontoxic. The Gillies group has also developed analogous self-immolative polymers based on a poly(glyoxylamide) backbone.^47^ As observed for the poly(glyoxylates), these new polymers disassemble from head to tail upon exposure to a specific stimulus that removes the terminal capping group. Interestingly, poly(glyoxylamide) exhibited thermal properties that differ from those of poly(glyoxylates). Such properties may be advantageous for certain polymer applications.

The Phillips group designed and synthesized a unique class of self-immolative polymer based on a poly(carboxypyrrole) backbone^48^ (Figure 5E). The poly(carboxypyrrole) disassembles through sequential 1,4-elimination and decarboxylation reactions. Diverse responsive groups, which can respond to various analytes such as hydrogen peroxide, were used to cap the polymer head. These polymers depolymerize completely from head to tail when they are triggered by specific applied signals that react with the responsive units. Remarkably, such poly(carboxypyrrole) self-immolative polymers disassemble in the solid state. This unique property offers a starting point for creating selectively depolymerizable coating materials.

Recently, Niu and co-workers reported a novel class of enyne self-immolative polymers that are capable of disassembly through a metathesis cascade triggered depolymerization^49^ (Figure 5F). The polymer backbone is composed of 1,6-enyne building block units, which are efficient substrates for a metathesis reaction.^50^ These self-immolative polymers have excellent stability in strong acid and base and nucleophiles and at elevated temperatures. Upon exposure to a metathesis catalyst, the polymers undergo efficient and complete sequential depolymerization.

## Polymers with Sequential Disassembly Mechanism Initiated from an Inner Site

The disassembly pathway that stands behind the original design of self-immolative polymers relies on the removal of a head trigger and sequential fragmentation along the polymer backbone. Degradable polymers have also been developed with disassembly mechanisms that are initiated from inner sites of the polymer backbones. In this section of this Perspective, we focus on selected examples of such degradable polymers.

**Figure 6:**
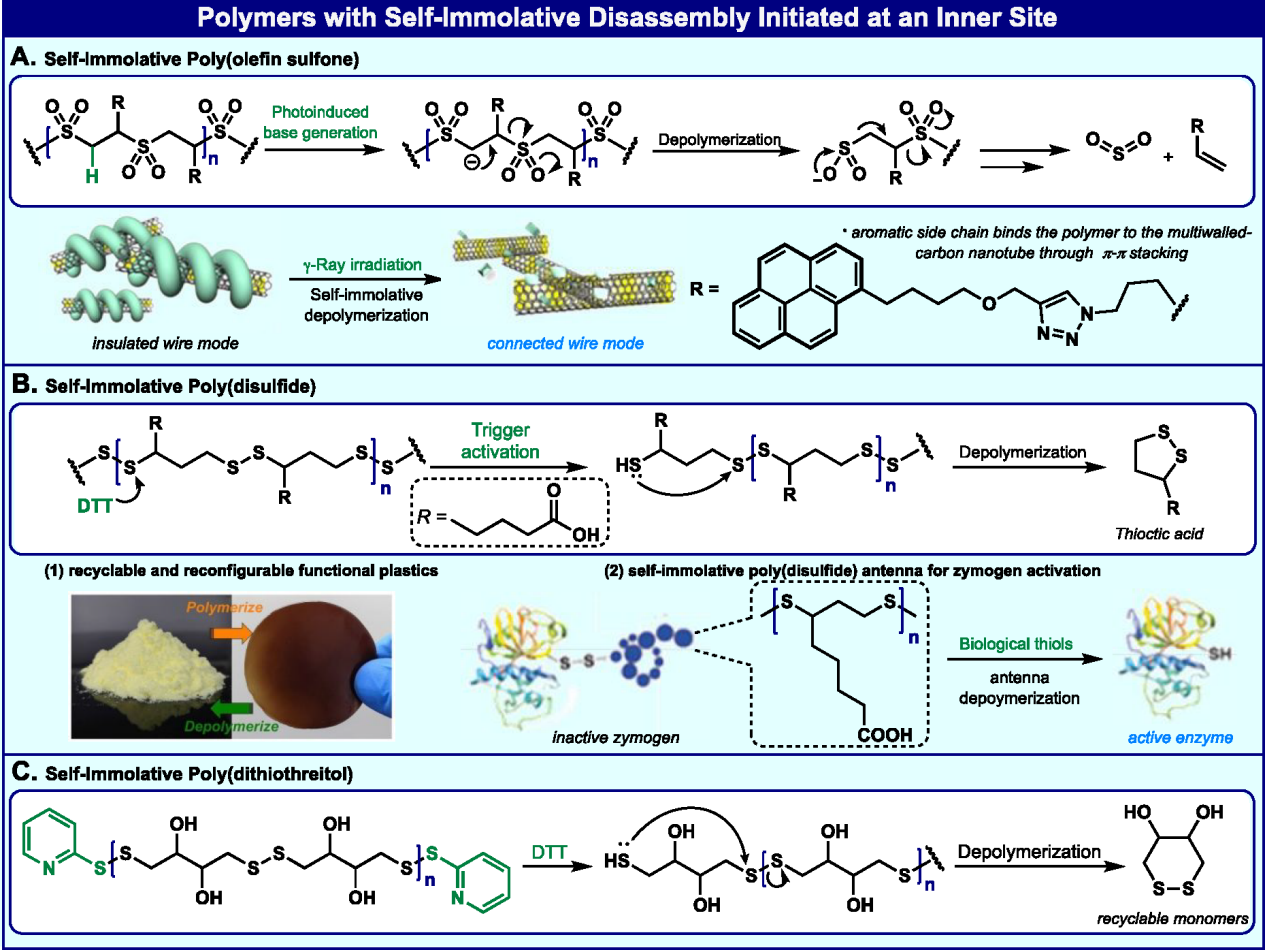
Selected examples of polymers with a sequential disassembly mechanism initiated from an inner site. Image (B.1) reproduced with permission from ref (55). Copyright 2021 Elsevier. Image (B.2) reproduced with permission from the author of ref (56).

The Sasaki group was among the first to develop polymers with a poly(olefin sulfone) backbone, equipped with photobase groups located internally^51^ (Figure 6A). Exposure of the polymer to UV light generated an amine base, which extracted protons from the main chain of the poly(olefin sulfone) and induced a depolymerization reaction of the polymer backbone. A visible lithographic image could be obtained from a polymer film, which was exposed to UV light and heat. Moore and co-workers reported a similar polymer with a poly(vinyl *tert*-butylcarbonate sulfone) backbone.^52^ They have shown that such polymers can thermally decompose via carbonate elimination as the rate-determining step.

Swager and co-workers synthesized poly(olefin sulfone) polymers bearing pyrene side groups and used them to wrap single-walled carbon nanotubes (SWCNTs) through aromatic π–π interactions.^53,54^ The resulting poly(olefin sulfone)-SWCNT composites serve as active transducers in a novel class of γ-ray dosimeters (Figure 6A). The polymer backbone was readily depolymerized on exposure to ionizing radiation. The polymer degradation results in immediate changes in the electronic potential of the device active layers.

Qu, Tian, and Feringa reported the development of a poly(disulfide) polymer that can disassemble via cyclization reactions upon exposure to base or reductive agents.^55^ Other groups have reported similar developments of degradable polymers based on the poly(disulfide) structure (Figure 6B). The reversible nature of the disulfide bond enables poly(disulfides) to be efficiently recycled, using a basic aqueous solution (Figure 6B.1). The properties of the materials are fully recovered after multiple cycles and have the potential for use as green plastics.

In a recent report, Zelikin and co-workers described the use of a poly(disulfide) polymer to successfully mask an active site of an enzyme to form an inactive zymogen^56^ (Figure 6B.2). The active site was reactivated upon exposure to biological thiols. The poly(disulfide) acts as a molecular antenna, which can receive cleavage signals at various sites along the polymer backbone and then undergo sequential disassembly to unmask the enzyme’s active site. Daasbjerg and co-workers reported self-immolative polymers with a new poly(disulfide) backbone, using dl-dithiothreitol (DTT) as the monomer. Poly(DTT) was produced by solid-state polymerization in a robust and easily scalable process by mechanically mixing DTT with 2,2′-dithiodipyridine as the end-capping agent (Figure 6C). These polymers undergo efficient depolymerization initiated by DTT. In subsequent work, Daasbjerg and co-workers showed that, by decorating the self-immolative poly(DTT) backbone with pendant catechol units, the polymer could be shaped into stimuli-responsive gels, generated through pH-dependent catecholato–metal ion cross-links. The gel degradation, resulting from sequential disassembly of the polymer backbone was visualized by the release of an encapsulated dye.^57,58^

## Miscellaneous Examples of Polymers with Related Self-Immolative Disassembly

The majority of examples associated with self-immolative polymers are based on polybenzylcatbamates, most likely due to the simplicity of the monomer budling blocks and the efficiency of the polymerization protocol. Therefore, numerous publications describing various applications with such self-immolative polymers have appeared during the past few years.^59−67^ In addition to the examples described so far in this Perspective, several other polymer backbones with related disassembly mechanism have recently been reported.^68−71^ Moreover, the progress of the field has inspired scientists to develop polymers that employ a self-immolative dendritic unit as a focal junction for disassembly. Such polymers usually carry a responsive group on their self-immolative dendritic unit, which is attached to two polymeric side chains. Removal of the responsive group by an analyte of interest initiates the polymer disassembly, through two consecutive quinone-methide elimination reactions. Almutairi and co-workers were the first to report such polymeric nanoparticles.^72^ They used a polyester backbone attached to a self-immolative dendritic unit, which is equipped with a boronic ester triggering group as a substrate for hydrogen peroxide. The polymer was formulated in the shape of a capsule that underwent disassembly upon exposure to reactive oxygen species. Shortly thereafter, the Cheng group reported similar polymeric structures that were composed of polycarbamates and polyester backbones attached to self-immolative dendritic units.^73^ The polymers are able to trap and release a dye through their disassembly pathway.

In later work, the same group reported a new polymeric material in which the responsive group of the self-immolative dendritic unit could be removed by exposure to UV light. The polymer backbone was composed of the anticancer drug 10-hydroxycamptothecin and a self-immolative dendritic unit, attached each to other in an alternating order.^74^ Upon irradiation with UV light, the polymer backbone disassembles and the drug molecules are released.

Amir and co-workers designed photoactivated self-immolative junctions that enables the transformation of nonresponsive polymers into photocleavable polymers.^75^ The polymers undergo split disassembly into two equally sized fragments on exposure to a specific stimulus. Interestingly, when the polymer was formulated into a thin film, the quinone-methide-based disassembly occurred in the solid phase.

## Summary and Outlook

The inspiration for the development of the disassembly chemistry, used to achieve the function of self-immolative polymers, first originated from the quinone-methide elimination reaction. This unique reaction enabled scientists to design and synthesize spacer molecules that are known as self-immolative linkers. Such molecules, usually based on 4-aminobenzyl alcohol or 4-hydroxybenzyl alcohol, can undergo a fragmentation process, which is initiated at one terminus site, to release a target molecule from the other terminus. Incorporation of 4-aminobenzyl alcohol as a building block unit in a polymer backbone results in a macromolecular linear structure that can disassemble from head to tail. Capping of the polymer head with a responsive group seals the polymer with a trigger, which can be activated upon exposure to specific extrenal stimuli.

The distinct disassembly profile of self-immolative polymers sets them apart from other degradable materials. Our original report of self-immolative polymers in 2008 has opened the door for a new class of stimuli-responsive disassembled materials. After this report, several groups from around the world developed new molecular building blocks that can be used to compose self-immolative polymers. Our group has mainly focused on the use of self-immolative polymers as tools to achieve effective signal amplification and controlled-release functions. Other groups have also developed applications for materials science and for biomedicine.

The growing interest in self-immolative polymers is clearly reflected by the increasing number of publications in this field. Remarkably, new examples of structures and applications of self-immolative polymers keep emerging even while these lines are being read. Ultimately, the development of new molecular backbones for self-immolative polymers with enhanced properties and disassembly behavior still remains a general challenge. The continuous demand for stimuli-responsive degradable materials guarantees the future interest of the scientific community in this unique class of polymers.
